# Systematic Hydration Rounds Versus a Socially Assistive Robot to Promote Fluid Intake in Institutionalized Older Adults: A Quasi-Experimental Study

**DOI:** 10.3390/nu18162715

**Published:** 2026-08-20

**Authors:** Cristina Vallès-Carvajal, Laia Selva-Pareja, Olga Masot, Maria Falcón-Villaitodo, Carla Camí, Teresa Botigué

**Affiliations:** 1 Department of Nursing and Physiotherapy, University of Lleida, 25198 Lleida, Spain; cristina.valles@udl.cat (C.V.-C.); olga.masot@udl.cat (O.M.); maria.falcon@udl.cat (M.F.-V.); carla.cami@udl.cat (C.C.); teresa.botigue@udl.cat (T.B.); 2 Biomedical Research Institute of Lleida—Dr. Pifarré Foundation, IRBLleida, 25198 Lleida, Spain; 3 Health Education, Nursing, Sustainability and Innovation Research Group (GREISI), Department of Nursing and Physiotherapy, University of Lleida, 25198 Lleida, Spain

**Keywords:** aged, nursing homes, dehydration, fluid intake, hydration monitoring, socially assistive robots

## Abstract

**Background:** Several strategies have been proposed to promote hydration among institutionalized older adults, including systematic hydration rounds and, more recently, the use of Socially Assistive Robots (SAR). However, their implementation in nursing homes remains limited. **Objective:** To compare changes in fluid intake over a one-month period among residents exposed to two hydration-promotion strategies and to identify baseline characteristics associated with fluid intake. **Methods:** A quasi-experimental study was conducted in a nursing home. Residents were assigned to either a systematic hydration rounds intervention with manual recording or a SAR-supported multicomponent educational intervention including reminders, positive reinforcement, and digital intake monitoring. Fluid intake was monitored for one month and analyzed using adjusted mixed-effects linear regression models. **Results:** Residents in the systematic hydration rounds group showed higher fluid intake throughout follow-up. In the longitudinal analysis, assignment to the SAR-supported group was independently associated with lower fluid intake during follow-up (β = −354.24 mL/day; *p* < 0.001), after adjustment for baseline fluid intake and functional dependence. No significant differences were observed in intake trajectories between strategies. Dysphagia was independently associated with lower fluid intake, whereas diabetes was associated with higher fluid intake. **Conclusions:** Residents assigned to the systematic hydration rounds group showed higher fluid intake during follow-up. Dysphagia was associated with lower fluid intake, highlighting the need to adapt hydration-promotion strategies to residents’ swallowing-related needs. Although the SAR-supported intervention was not associated with higher fluid intake, the findings suggest that technological tools for hydration promotion and monitoring require adequate integration into routine clinical practice.

## 1. Introduction

Dehydration due to low fluid intake is a common condition of high clinical relevance among older adults living in nursing homes. A recent meta-analysis [1] estimated a prevalence of 34% using serum osmolality >300 mOsm/kg, considered the most reliable measure for its identification [2]. Dehydration is associated with worse health outcomes, including an increase in morbidity and mortality, a higher risk of suffering from clinical complications, and more hospital admissions [3]. Despite its prevalence, consequences, and impact on healthcare costs being well-documented, dehydration remains a persistent problem, making interventions in nursing homes necessary [4].

To prevent and address dehydration, it is essential to ensure an adequate fluid intake that is adapted to individual preferences and needs. In this regard, the European Society for Clinical Nutrition and Metabolism (ESPEN) [2] recommends a minimum intake of 1.6 L for women and 2.0 L for men older than 65 years, except in cases of clinical contraindications. However, low fluid intake remains common in nursing homes, with prevalence rates ranging from 24% to 85.5% for fluid intakes below 1500 mL/day [5,6,7].

This low intake can be explained, in part, by individual factors of the residents themselves [8,9]. Among them, reduced thirst sensation and memory problems associated with age can influence fluid intake. These factors can contribute to a refusal to drink, either because the need is not perceived, or because they do not remember that they have not been drinking [10]. In this regard, behavioral strategies have shown their potential for promoting intake and addressing rejection. These strategies include systematic hydration reminders [3,8,11], visual cues [11], social drinking, positive reinforcement, and social activities [8,11]. Among them, the frequent and active offering of drinks, such as through hydration rounds, has shown promising results for fluid intake among nursing home residents [12,13,14,15,16].

In this context, to assess the effectiveness of these strategies, it is essential to systematically monitor fluid intake [4]. In clinical practice, self-reported fluid intake diaries are considered the most recommended method for monitoring fluid intake due to their accuracy, which is comparable to direct observation. However, their use is limited to residents who are able to collaborate [17]. For this reason, fluid intake records completed by care staff are commonly used. Despite this, these records have not been widely implemented, or their use is restricted to specific residents [18]. Moreover, the volume in milliliters and the characteristics of the drinks are not always specified [8], and records can be affected by staff’s tendency to overestimate actual intake [19,20]. These difficulties are further compounded by time constraints and high care workload, which hinder record completion [8]. Taken together, these limitations reflect organizational and structural barriers that hinder both the promotion and monitoring of hydration in daily practice [8]. As an alternative, digital recording tools have been proposed as a promising strategy for the active monitoring of hydration, as they can be more accurate than paper-based records [21]. Accordingly, it is necessary to develop digital fluid intake recording systems that are more accurate, effective, and easily integrated into clinical routines. Most previous studies evaluating hydration-promotion strategies assessed fluid intake only at pre- and post-intervention [12,16] or over short observation periods [13,14,15]. Therefore, continuous monitoring throughout the intervention period, using a recording system that addresses the limitations of current methods, may provide a more comprehensive evaluation of temporal changes in fluid intake.

In response to these challenges, Socially Assistive Robots (SAR) emerge as a potentially useful technology in nursing homes. This is because they can facilitate routine and repetitive tasks, such as monitoring [22,23], thereby reducing stress and care workload and allowing care staff to dedicate more time to individualized and empathetic care [23]. Likewise, they can stimulate communication, social interaction, and companionship among older adults, promoting their acceptance [22,23]. In this way, SARs could support hydration management by addressing both resident-related barriers to adequate fluid intake and the practical challenges of continuously monitoring hydration in nursing homes. Functionalities such as voice-driven interaction, touch interfaces, and the possibility of offering personalized stimuli would allow implementing behavioral strategies, such as reminders or directed activities [24]. In addition, SARs can incorporate specific systems of digital recording [24], suggesting their potential to support structured monitoring of fluid intake. Despite this potential, the effectiveness of SARs in hydration care remains largely unexplored. Existing evidence is limited to technological prototypes developed in community settings that incorporate hydration reminders or prompts to drink [25,26,27]. However, none of these systems have been clinically evaluated for their effect on daily fluid intake, and the use of SARs to support continuous digital monitoring of fluid intake has not yet been investigated.

Evidence is therefore needed regarding the potential role of SARs in supporting both hydration promotion and structured monitoring of fluid intake in nursing homes. In addition, limited information is available on how such approaches compare with structured hydration strategies already embedded in routine care, such as systematic hydration rounds. Evaluating these approaches through continuous daily monitoring of fluid intake may contribute to a better understanding of their potential role in supporting hydration care in long-term care facilities. Accordingly, the aim of the present study was to evaluate and compare the evolution of daily fluid intake over a one-month period among institutionalized older adults exposed to two hydration-promotion strategies, one based on systematic hydration rounds and the other on a SAR-supported multicomponent educational intervention, and to analyze residents’ baseline characteristics associated with fluid intake.

## 2. Materials and Methods

### 2.1. Study Design

A comparative quasi-experimental study was conducted with two intervention groups in a nursing home over a one-month follow-up period (July 2024). This duration was consistent with previous hydration interventions in nursing homes, in which changes in fluid intake and hydration status had been observed over periods of four to eight weeks [16,28,29,30]. The study compared a SAR-supported multicomponent educational intervention with the systematic hydration rounds routinely implemented in the nursing home.

### 2.2. Context and Participants

The study was performed in a nursing home for adults older than 65 years of age in Lleida (Spain). This nursing home has a capacity of 60 residents across four floors, with 15 residents per floor. All the residents were invited to participate, except those receiving end-of-life care.

### 2.3. Interventions

Two interventions aimed at promoting residents’ fluid intake and improving daily recording were analyzed and compared. The first intervention corresponded to the center’s usual care procedure. This procedure consisted of six systematic hydration rounds distributed throughout the day: breakfast (9 a.m.), mid-morning (11 a.m.), lunch (1 p.m.), afternoon snack (4 p.m.), late afternoon (6 p.m.), and dinner (8 p.m.). During these rounds, the care staff distributed the drinks, reminded and encouraged residents to drink, respected their individual preferences, and provided assistance with drinking when needed. A previous qualitative study conducted in the same center [8] showed that the fluid intake was usually recorded dichotomously, simply indicating whether the resident had drunk or not. For this purpose, during the follow-up period, a specific paper-based record was added to quantify the daily fluid intake according to the time of day, the type of drink, and the texture for each participant.

The second intervention consisted of a multicomponent educational intervention on hydration, delivered by a SAR. SARs are automated interactive robots with sensors for smart navigation, a touchscreen, camera, microphone, and speakers. These characteristics allow them to navigate throughout the unit, present audiovisual content, and interact with the residents and care staff through voice and a touch interface. More specifically, these were TEMI social robots incorporating the SOMCare smart care platform designed by the Saltó Group (Lleida, Spain) [31].

**Figure 1:**
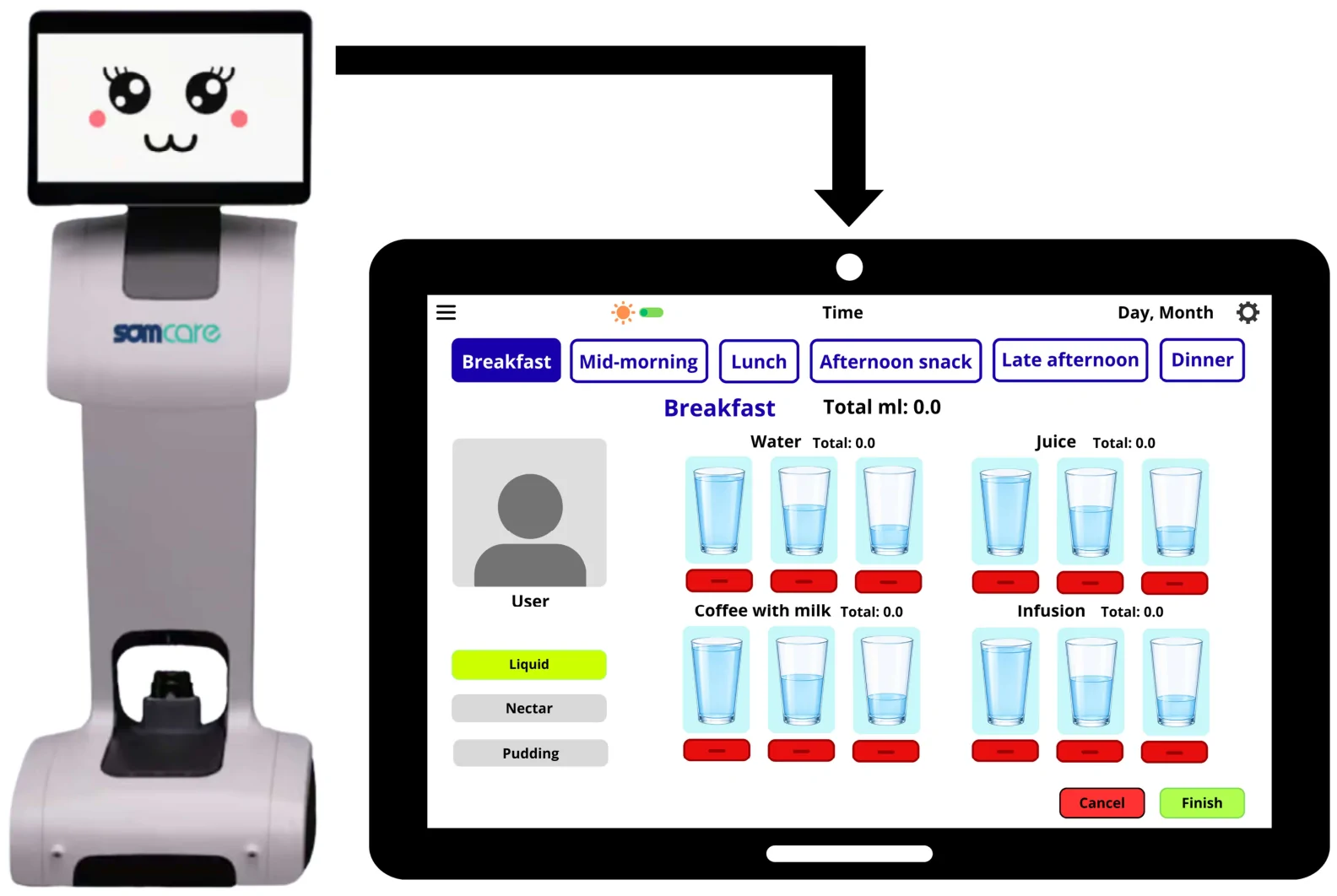
SAR and digital fluid intake recording system. As an example, the screen corresponding to the breakfast period is shown. At the top of the screen are the six daily recording periods, with the current period highlighted. For each resident, the prescribed liquid texture was preselected, although it could be changed if necessary. The screen displayed the beverages offered during the selected period. For each beverage, three icons represented a full serving, a half serving, and a quarter serving of the container; pressing them in succession allowed the consumed volume to be recorded, while the “−” button subtracted the corresponding amount. Both the volume consumed for each drink and the total fluid intake (mL) were updated in real time. Selecting “finish” saved the record and automatically moved the application to the next resident, while “cancel” cleared the recorded amounts.

In the SAR-supported intervention, the beverages offered and the six hydration time slots were identical to those in the usual care procedure, and the care staff continued to handle the distribution of the beverages, respecting the residents’ individual preferences and offering them assistance during intake. The difference between the two interventions was that reminders, which had previously been provided by the care staff, were now delivered by the SAR, which also incorporated positive reinforcement and a digital fluid intake recording system. Each day, the SAR moved autonomously to the designated resident or professional, according to the programmed activity. The SAR delivered six daily reminders, coinciding with the established time slots: in each of them, it first encouraged the residents to drink and then reminded the care staff to offer the drinks and record the intake. The resident–robot interaction was primarily receptive, although residents could respond verbally, receiving standardized responses that encouraged them to drink. They could also use the touchscreen to make requests to the robot, such as checking information or playing supportive audiovisual content, including educational and motivational materials related to hydration. In addition, the SAR included a digital fluid intake recording system used exclusively by the care staff. This system allowed the same variables collected in the paper-based record (time of intake, type of beverage, amount ingested, and texture of the administered liquid) to be documented for each resident, but via a digital, real-time interface (Figure 1).

After each reminder, the SAR approached the care staff, reminded them to complete the fluid intake record, automatically opened the record corresponding to that time slot, and did not return to the charging base until all records for that period had been completed, thereby promoting the completion of digital records throughout the intervention. Additionally, personalized positive reinforcement was applied twice a day (in the morning and in the afternoon): the SAR compared each resident’s recorded intake with the daily threshold of 1500 mL, sending congratulatory messages to those who met it and motivational messages to those who did not. Before the intervention, the care staff received a brief training session (approximately one hour) on how the SAR and the recording interface worked.

Throughout the intervention period, a member of the research team monitored the proper implementation of the interventions and recording procedures in both groups. This supervision included daily verification of the completion of paper-based and digital fluid intake records, as well as monitoring the operation of the SAR system. Daily monitoring confirmed the completion of all scheduled fluid intake records in both groups throughout each participant’s follow-up period. Technical issues were resolved by the technician from the robot’s supplier company. When a hydration reminder, positive reinforcement message, or other scheduled activity was found not to have run correctly due to technical or programming issues, it was rescheduled to ensure it was completed. Consequently, all scheduled SAR reminders, positive reinforcement messages, and other programmed activities were ultimately delivered as intended. Resident-level adherence to the resident-facing components of either intervention, such as whether residents drank in response to reminders or encouragement, was not assessed.

Given that the center was divided into four floors, the SAR-supported intervention was performed on two randomly selected floors, while respecting the usual distribution of residents. The other two floors maintained the systematic hydration rounds and the paper-based fluid intake records. This design allowed the usual logistics and routine of the center to be maintained while avoiding the risk of contamination among participants. Although the intervention was implemented at floor level, the four units did not operate as independent care settings. All floors followed the same standardized care protocols and shared common nursing supervision and management structures. Consequently, hydration-related practices and care procedures were guided by the same institutional criteria across the facility. The intervention period lasted one month.

### 2.4. Variables and Instruments

The sociodemographic variables were age and gender. Clinical variables included functional status measured with the Barthel Index [32], nutritional status measured with the Mini Nutritional Assessment Short Form (MNA-SF) [33], and the risk of pressure ulcers assessed using the Braden Scale [34]. The presence of urinary incontinence was recorded, and dysphagia was assessed using the Volume-Viscosity Swallow Test (V-VST) [35]. With respect to diseases, the following were taken into consideration: diabetes, congestive heart failure, diagnosed dementia, stroke, kidney disease, and the presence of more than four chronic diseases. Lastly, the presence of polypharmacy, defined as the use of ≥5 medicines [36], was also considered, and dehydration was assessed using serum osmolality, with values >300 mOsm/kg considered indicative of dehydration [2].

In addition, the daily fluid intake was recorded for each participant. In both groups, the same variables were collected: time of day (breakfast, mid-morning, lunch, afternoon snack, late afternoon, and dinner), type of fluid (water, juice, coffee, coffee with milk, infusion, or others), amount ingested in milliliters, and texture of the fluid (liquid, nectar, or pudding). The quantity ingested was estimated in milliliters based on the proportion consumed from the container, i.e., in its entirety, three-quarters, half, or a quarter of the volume of the container used for each type of drink.

### 2.5. Data Collection

The study variables were assessed at two time points, before and after the intervention, while fluid intake was recorded continuously throughout the follow-up period. To estimate baseline fluid intake, each resident’s fluid intake was recorded during the three days preceding the intervention using the recording system assigned to the corresponding intervention group.

The sociodemographic and clinical variables were collected through an ad hoc questionnaire, which included the corresponding scales. These data were collected by previously trained researchers to guarantee the homogeneity and standardization of the data collection procedures.

In addition, fluid intake was recorded through different procedures according to the intervention. In intervention 1, a specific paper-based recording form was used, while in intervention 2, the digital recording system integrated into the SAR was used. In both interventions, the recording was performed by the center’s geriatric care workers, because they provided the main support for residents’ hydration. To this end, they received specific training on how to record fluid intake, and in the case of the SAR, a pilot test was conducted to test its usability and feasibility.

### 2.6. Data Analysis

A descriptive analysis of the characteristics of the sample was performed. Qualitative variables were described using absolute frequencies and percentages, and the quantitative variables were described using means and standard deviations or medians and interquartile ranges, as appropriate, after verifying the distribution of the variables.

No data were missing for baseline sociodemographic or clinical variables. Two participants did not complete the follow-up period. All fluid intake observations collected until the end of their participation were retained in the longitudinal analyses, and mixed-effects models were fitted using all available repeated measurements. Individual and group trends in fluid intake throughout the study period were graphically represented using LOESS-smoothed trends and their corresponding 95% confidence intervals. Mean fluid intake at baseline and during the intervention period was analyzed within each of the intervention groups.

Differences in the evolution of fluid intake during the study period between intervention groups were analyzed through mixed-effects linear regression models including the participant as a random effect and intervention group, time (day of intervention, centered to the last day), their interaction, and baseline fluid intake (centered to its mean) as fixed effects. Additionally, the models were adjusted for baseline resident characteristics considered potentially relevant as confounding factors in the relationship between intervention exposure and fluid intake. Given the available sample size, the number of covariates included in the models was intentionally kept limited to reduce the risk of overfitting and preserve the stability of the model estimates.

The predictive contribution of other baseline characteristics potentially associated with fluid intake, previously classified into two categories, was assessed by adding each characteristic individually as an additional term to the main mixed-effects regression model. Finally, all baseline characteristics showing a significant association with changes in fluid intake in these individual analyses were included simultaneously in a final mixed-effects linear regression model.

The marginal and conditional coefficients of determination (R^2^), the variance components of the random effects, and the intraclass correlation coefficient (ICC) were estimated for each model. The statistical analysis was performed with the R software (version 4.5.3) [37], and the level of significance was set at *p* < 0.05.

### 2.7. Ethical Considerations

The study was authorized by the center and approved by the Research Ethics Committee for drugs at the Arnau de Vilanova University Hospital, Lleida Territorial Management—GSS, Spain (CEIC-3054, 22 April 2024). It was conducted in accordance with the principles of the Declaration of Helsinki and current data protection regulations.

All of the participants, or their legal representatives where applicable, received information about the aims of the study, the implications of their participation, data processing, and their rights through an information sheet. Subsequently, written, informed, and voluntary consent was obtained from each participant or legal representative, where applicable. All residents included in the study agreed to participate, and no instances of refusal to participate or to interact with the robot were observed throughout the intervention. The right to withdraw from the study at any time without any impact on the care received was guaranteed.

All the data collected were treated confidentially and pseudonymized using alphanumeric identifiers, with access restricted exclusively to the authorized research team. Although the SAR was equipped with a camera, microphone, sensors, and autonomous navigation, no audio or video data were recorded or stored, and the sensors were used exclusively for real-time navigation, without data storage. The only data recorded by the SAR were the residents’ daily fluid intake, entered manually by the care staff via the touchscreen. To facilitate identification, the screen displayed a photograph of each resident, obtained with prior consent and used exclusively for this purpose. The data were stored securely and managed in accordance with applicable data protection regulations.

## 3. Results

### 3.1. Sample Characteristics

A total of 57 residents participated in the study, of whom 55 completed the intervention period. Overall, most participants were women (61.4%), and the mean age was 85.2 ± 7.54 years. Table 1 shows the remaining baseline characteristics of the sample and differences between intervention groups. The sample was characterized by advanced age, as more than half of the residents were ≥85 years old. Likewise, high clinical and functional complexity was observed, with 66.7% of participants diagnosed with dementia, 59.6% with functional dependence, 33.3% with dysphagia, 57.9% with a risk of developing pressure ulcers, and almost the entire sample presenting four or more chronic diseases and polypharmacy. In general, the baseline characteristics were comparable between groups, except for the functional status, in which functional dependence was higher in intervention group 2 (*p* = 0.023).

### 3.2. Fluid Intake and Temporal Trends by Intervention Group

Table 2 shows fluid intake at baseline and during the intervention period in the overall sample and by intervention group. Overall, a slight decrease in the mean fluid intake was observed during the intervention period compared with baseline. Nevertheless, significant differences were identified between groups at both baseline and during the intervention, with a higher intake in intervention group 1 than in intervention group 2 (1784 vs. 1527 mL/day; *p* = 0.020 and 1749 vs. 1329 mL/day; *p* < 0.001, respectively).

When analyzing the intake by time of day, intervention group 1 also showed a higher intake during the morning at both baseline (937 vs. 738 mL/day; *p* = 0.006) and during the intervention period (959 vs. 692 mL/day; *p* < 0.001). In the afternoon, no significant differences were observed between groups at baseline. However, during the intervention period, fluid intake was significantly higher in intervention group 1 than in intervention group 2 (790 vs. 637 mL/day; *p* = 0.001).

**Figure 2:**
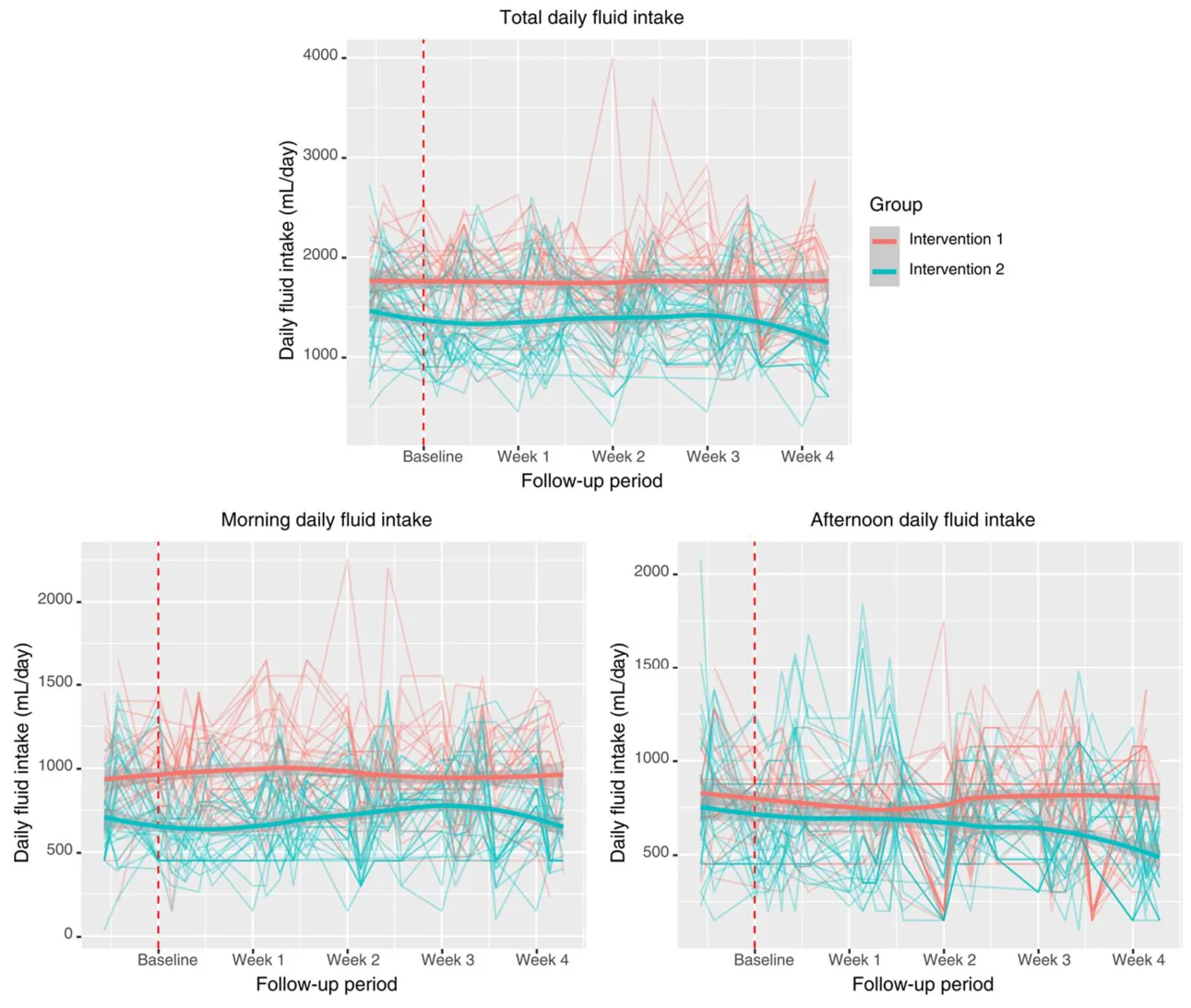
Trends in mean total daily fluid intake and intake by time of day during the intervention period, according to intervention group. The shaded areas represent the 95% confidence intervals of the LOESS-smoothed trend. The red dashed lines indicate the time point separating the baseline fluid intake recording period from the intervention period.

In addition, Figure 2 shows temporal trends in mean total daily fluid intake and intake by time of day throughout the intervention period in both groups. Overall, intervention group 1 showed a relatively stable intake during the entire period, while intervention group 2 showed a decreasing trend, especially in total intake and during the afternoon.

### 3.3. Longitudinal Analysis of Changes in Fluid Intake

Table 3 shows the results of the mixed-effects linear regression model used to analyze changes in fluid intake during the study period. The model included intervention group, time, their interaction, and baseline fluid intake as fixed effects; participant as a random effect; and was additionally adjusted for functional dependence because it was the only baseline clinical characteristic showing between-group differences and was therefore considered a potential source of confounding in the longitudinal analyses.

No significant interaction between group and time was observed, nor was there a significant effect of time. Functional dependence also did not show a significant independent contribution to the model. In contrast, baseline fluid intake (β = –0.56; 95% CI: −0.70 to −0.42; *p* < 0.001) and intervention group were significantly associated with changes in fluid intake during follow-up. Specifically, intervention group 2 had a significantly lower fluid intake than intervention group 1 (β = −354.24 mL/day; 95% CI: −485.61 to −222.93; *p* < 0.001).

### 3.4. Baseline Characteristics Associated with Changes in Fluid Intake

To identify the baseline characteristics associated with changes in fluid intake during follow-up, each of the clinical variables considered was individually incorporated into the linear mixed-effects regression model described above. Of the variables analyzed, only dysphagia and diabetes showed a significant association with changes in fluid intake during follow-up (Table 4).

These variables were subsequently incorporated into a final model along with the variables included in the main model. As shown in Table 5, after simultaneously considering all variables included in the model, dysphagia was associated with a lower fluid intake during follow-up (β = −230.55; 95% CI: –352.29 to –108.97; *p* < 0.001), whereas diabetes was associated with higher fluid intake (β = 125.08; 95% CI: 23.96 to 226.16; *p* = 0.014).

Baseline fluid intake and the intervention group continued to show a significant association with the changes observed during follow-up. In contrast, neither functional dependence, time, nor the group × time interaction showed a significant independent contribution to the final model.

## 4. Discussion

The present study compared changes in fluid intake over a one-month period among institutionalized older adults exposed to two hydration-promotion strategies. Overall, descriptive analyses showed higher and relatively more stable fluid intake in the systematic hydration rounds group, whereas the group receiving the SAR-supported multicomponent educational intervention, which incorporated reminders, positive reinforcement, and a digital recording system, showed a generally declining intake trend, particularly during the afternoon. However, the interpretation of these differences requires consideration of the complexity of the factors influencing fluid intake among nursing home residents, a multifactorial issue affected by both individual and organizational factors [7,8,38]. Therefore, the interpretation of the findings requires consideration of the specific characteristics of the implemented strategies, the procedures used to monitor fluid intake, and the residents’ clinical profile, as these factors may have influenced the observed differences.

The trends observed throughout the follow-up period provide complementary information to that typically reported in hydration intervention studies, which are often limited to comparisons between baseline and post-intervention measurements or to relatively short observation periods [12,13,14,15,16]. In this regard, continuous monitoring of fluid intake made it possible to characterize temporal changes in intake and to identify variations in drinking patterns throughout the day. This approach is particularly relevant in the nursing home setting, where opportunities for fluid intake, levels of supervision, and residents’ needs may vary over the course of the day. However, these descriptive observations should be interpreted with caution, as the longitudinal analysis did not identify a significant group × time interaction. This finding indicates that the visual differences observed in intake trajectories were not reflected in statistically different temporal patterns between the two strategies during the study period.

Another important consideration when interpreting the findings is that the two groups did not start with equivalent baseline conditions. Specifically, the group receiving the SAR-supported intervention had lower baseline fluid intake and a higher proportion of residents with functional dependence. In this regard, the longitudinal analysis showed that baseline fluid intake was significantly associated with changes observed during follow-up, highlighting the importance of considering residents’ initial status when evaluating changes in fluid intake over time. Nevertheless, differences between groups remained after adjustment for baseline fluid intake and functional dependence, whereas the latter did not show a significant independent contribution to the model. Overall, these findings suggest that baseline differences between groups may have contributed to the observed results. However, the adjusted analyses indicate that the observed between-group differences during follow-up cannot be fully attributed to the baseline variables included in the model.

Regarding the implemented strategies, it is important to note that both interventions incorporated several components that have been identified in the literature as relevant for promoting fluid intake among institutionalized older adults. These include frequent drinking opportunities and the active offering of fluids throughout the day, which are considered key elements for maintaining adequate hydration [3,11]. Likewise, drinking reminders, respect for individual preferences, and assistance during fluid intake have been described as potentially useful strategies for promoting fluid consumption, particularly among individuals with greater functional dependence or a higher need for assistance [13,14]. The frequency of drinking opportunities also appears to be important, and it has been suggested that offering between six and eight opportunities to drink throughout the day may contribute to the maintenance of adequate hydration [29]. In addition, the integration of beverage distribution and assistance with drinking into usual care activities has been identified as a facilitating factor for the implementation of these strategies [15,30]. In our study, systematic hydration rounds were part of the usual care embedded within the organizational routine of the nursing home, and relatively stable fluid intake levels were observed throughout the follow-up period in this group. Although integration into routine care may represent a plausible explanation for this finding, this potential contribution could not be directly assessed in the present study. Despite sharing these common elements, the SAR-supported intervention incorporated additional components, including automated drinking reminders, positive reinforcement, and educational content on hydration. These features are consistent with behavioral and multicomponent strategies that have been identified in the literature as potentially useful for promoting fluid intake among older adults [3,11,39]. However, interventions of this type often require greater coordination, resources, and integration into usual care workflows to be sustained effectively over time [40]. This consideration is particularly relevant in the case of SARs, as several studies have highlighted that their contribution depends not only on their technological features but also on how they are integrated into care processes and usual clinical practice [23,24,41].

Given the multicomponent nature of the intervention, the observed findings reflect the combined implementation of its different components. However, given the quasi-experimental design, the mechanisms underlying the observed between-group differences cannot be determined, and the specific contribution of each intervention component cannot be assessed separately. Among the components incorporated into the intervention, the fluid intake recording system deserves particular consideration. A previous qualitative study conducted in the same facility found that fluid intake was not recorded specifically but rather documented only through dichotomous indicators of consumption [8]. For this reason, more detailed recording systems were implemented in both groups in the present study, although the documentation procedures were not equivalent. Whereas the systematic hydration rounds group used a structured paper-based record that was typically completed at the end of each shift, the SAR-supported intervention incorporated a real-time digital recording system that required fluid intake to be documented after each hydration round. Previous studies have identified documentation processes, their integration into care workflows, and the use of digital technologies as factors that may influence the quality and completeness of clinical records [42,43,44]. In addition, the digital recording system may have facilitated a more standardized data collection process and recorded closer to the actual time of consumption, as previously described [21]. However, the greater level of detail required by the digital recording system may also have resulted in a more demanding documentation process for staff. Fluid intake records may also be affected by limitations in accuracy, reliability, and objectivity, as it has been reported that staff tend to record beverages offered rather than those consumed by residents [17,18,45,46]. Furthermore, some authors have suggested that when the same staff members who implement hydration rounds are also responsible for recording fluid intake, the effects of the intervention may be overestimated [12]. These factors should be considered when interpreting the findings, as they may have influenced the quality and accuracy of fluid intake records. Consequently, it is not possible to determine the extent to which the observed differences reflect actual differences in fluid intake, differences arising from the documentation procedures, or a combination of both factors. Therefore, the observed association between intervention group and fluid intake should be interpreted with caution.

Beyond the differences observed between interventions, the findings indicate that certain baseline resident characteristics may also influence fluid intake during follow-up. In the final model, dysphagia was independently associated with lower fluid intake, consistent with previous studies conducted among institutionalized older adults [6,7]. From a clinical perspective, the need for texture modification of liquids, the lower acceptability of some thickened fluid preparations, and the requirement for supervision or assistance during drinking have been associated with lower fluid intake among residents with dysphagia [2,47]. In contrast, residents with diabetes showed higher fluid intake during follow-up. Although this association has been less extensively studied in institutionalized populations, the previous literature has suggested that it may be related to physiological mechanisms involved in the regulation of thirst and fluid balance [48]. The persistence of the association observed for dysphagia reinforces the importance of systematically assessing swallowing difficulties when designing hydration-promotion strategies in nursing homes. Future research should explore how standardized nursing assessments can contribute to the identification of hydration-related needs in institutionalized older adults. Within comprehensive geriatric assessment frameworks, these assessments may support the development of individualized hydration strategies adapted to residents’ characteristics.

### Limitations

This study has some limitations that should be considered. First, the quasi-experimental design, although it helped preserve the usual care organization and reduce the risk of contamination between groups, could have introduced uncontrolled baseline differences that may have influenced the results. Future studies using randomized controlled designs would help confirm these findings.

Another methodological consideration is that the intervention was allocated at the floor level, whereas the analyses were conducted at the individual resident level. Although all floors shared standardized care protocols and a common management and nursing supervision structure, a possible correlation among residents within the same floor cannot be completely excluded. Given the limited number of floors included, multilevel or cluster-adjusted analyses were not considered appropriate. Therefore, this potential intrafloor correlation should be considered when interpreting the results.

In addition, the intervention period was relatively short, which limits the assessment of the sustainability of the observed effect in the medium and long term, particularly regarding the integration of SARs into usual clinical practice. Future studies should therefore evaluate whether the observed effects are maintained over longer implementation periods, as previously reported for other hydration-promotion interventions [14,15,46]. Nevertheless, fluid intake was recorded throughout the entire intervention month. This is uncommon in the available literature, where assessments are generally based on one-time measurements or shorter observation periods [6,7,16,17,46].

Likewise, fluid intake was assessed through staff records, which may have introduced information bias, including the potential overestimation of actual consumption. In addition, the fact that staff in the systematic hydration rounds group were aware that their strategy was being compared with an innovative SAR-supported intervention may have increased their attention to both hydration practices and recording procedures, giving rise to a possible Hawthorne effect [49]. Furthermore, the use of different recording procedures across intervention groups may have influenced the observed findings. Although both interventions implemented more detailed fluid intake recording procedures than those routinely used in the facility, differences in the timing and structure of the paper-based and digital recording procedures may have affected the completeness and accuracy of the recorded information. Because the intervention and documentation procedures differed simultaneously between groups, their independent contributions to the observed between-group differences cannot be disentangled. Therefore, it is not possible to determine the extent to which the observed differences reflect actual differences in fluid intake, differences arising from the documentation procedures, or a combination of both factors. This circumstance should be considered a potential source of measurement bias when interpreting the findings.

Finally, the relatively small sample size and recruitment from a single nursing home may limit the generalizability of the results to other settings with different organizational characteristics, care practices, and resident profiles.

## 5. Conclusions

Residents assigned to the systematic hydration rounds group showed higher fluid intake during the follow-up period than those assigned to the SAR-supported multicomponent educational intervention, and this association remained significant after adjustment for baseline fluid intake and functional dependence. However, these findings should be interpreted with caution, as differences in the fluid intake recording procedures between groups may have influenced the observed results, and the specific contribution of this factor cannot be determined within the present study design.

Dysphagia was independently associated with lower fluid intake during follow-up, highlighting the importance of adapting hydration-promotion strategies to residents’ clinical needs, particularly in the presence of swallowing difficulties.

Although the SAR-supported intervention was not associated with higher fluid intake in this context, the findings suggest that the incorporation of technological tools to promote and monitor hydration may require adequate integration into routine clinical practice to maximize their potential. However, the present design does not allow the mechanisms underlying the observed between-group differences or the contribution of individual intervention components to be determined. Future research should use designs that allow the specific contributions of organizational, behavioral, and technological components to hydration outcomes and implementation to be assessed, while also examining how these components can be effectively integrated to support hydration within daily clinical practice.

## Figures and Tables

**Table 1:** Baseline characteristics of participants by intervention group.

Characteristics		Overall *n* = 57	Group		*p*-Value
			Intervention 1 *	Intervention 2 **	
Age	<85 years	24 (42.1%)	11 (39.3%)	13 (44.8%)	0.877
	≥85 years	33 (57.9%)	17 (60.7%)	16 (55.2%)	
Gender	Man	22 (38.6%)	11 (39.3%)	11 (37.9%)	1.000
	Woman	35 (61.4%)	17 (60.7%)	18 (62.1%)	
Functional status	Independent	23 (40.4%)	16 (57.1%)	7 (24.1%)	**0.023**
	Dependent	34 (59.6%)	12 (42.9%)	22 (75.9%)	
Nutritional status	No malnutrition	46 (80.7%)	25 (89.3%)	21 (72.4%)	0.201
	Malnutrition	11 (19.3%)	3 (10.7%)	8 (27.6%)	
Pressure ulcers	No risk	24 (42.1%)	14 (50.0%)	10 (34.5%)	0.359
	Risk	33 (57.9%)	14 (50.0%)	19 (65.5%)	
Urinary incontinence	No	6 (10.5%)	4 (14.3%)	2 (6.9%)	0.423
	Yes	51 (89.5%)	24 (85.7%)	27 (93.1%)	
Dysphagia	No	38 (66.7%)	20 (71.4%)	18 (62.1%)	0.640
	Yes	19 (33.3%)	8 (28.6%)	11 (37.9%)	
Diabetes	No	37 (64.9%)	16 (57.1%)	21 (72.4%)	0.352
	Yes	20 (35.1%)	12 (42.9%)	8 (27.6%)	
Congestive heart failure	No	50 (87.7%)	23 (82.1%)	27 (93.1%)	0.253
	Yes	7 (12.3%)	5 (17.9%)	2 (6.9%)	
Diagnosed dementia	No	19 (33.3%)	13 (46.4%)	6 (20.7%)	0.075
	Yes	38 (66.7%)	15 (53.6%)	23 (79.3%)	
Stroke	No	46 (80.7%)	22 (78.6%)	24 (82.8%)	0.948
	Yes	11 (19.3%)	6 (21.4%)	5 (17.2%)	
Kidney disease	No	41 (71.9%)	19 (67.9%)	22 (75.9%)	0.706
	Yes	16 (28.1%)	9 (32.1%)	7 (24.1%)	
≥4 chronic diseases	No	5 (8.8%)	2 (7.1%)	3 (10.3%)	1.000
	Yes	52 (91.2%)	26 (92.9%)	26 (89.7%)	
Polypharmacy	No	5 (8.8%)	1 (3.6%)	4 (13.8%)	0.352
	Yes	52 (91.2%)	27 (96.4%)	25 (86.2%)	
Dehydration	No	53 (93.0%)	25 (89.3%)	28 (96.6%)	0.604
	Yes	4 (7.0%)	3 (10.7%)	1 (3.5%)	

* Intervention 1: Systematic hydration rounds and paper-based fluid intake recording (*n* = 28); ** Intervention 2: SAR-supported multicomponent educational intervention and digital fluid intake recording (*n* = 29).

**Table 2:** Baseline daily fluid intake (*n* = 57) and during the intervention (*n* = 55) in the total sample and according to intervention group.

Fluid Intake Period	Time of Data Collection	Overall Mean (SD)	Group		*p*-Value
			Intervention 1 Mean (SD)	Intervention 2 Mean (SD)	
Total daily fluid intake	Baseline	1653 (421)	1784 (378)	1527 (428)	**0.020**
	Intervention period	1538 (355)	1749 (285)	1329 (200)	**<0.001**
Morning intake	Baseline	836 (275)	937 (286)	738 (228)	**0.006**
	Intervention period	825 (210)	959 (182)	692 (140)	**<0.001**
Afternoon intake	Baseline	818 (279)	847 (260)	789 (298)	0.436
	Intervention period	713 (171)	790 (125)	637 (181)	**0.001**

Values are presented in mL/day; SD: standard deviation.

**Table 3:** Adjusted mixed-effects linear regression model for changes in fluid intake during the study period.

Predictors	Estimate (β)	95% CI	*p*-Value
(Intercept)	23.40	[−84.73, 131.53]	0.668
Group [Intervention 2]	−354.24	[−485.61, −222.93]	**<0.001**
Time	−0.41	[−4.16, 3.33]	0.828
Baseline fluid intake	−0.56	[−0.70, −0.42]	**<0.001**
Functional dependence	−70.92	[−194.59, 52.66]	0.253
Group [Intervention 2] × Time	−3.27	[−8.22, 1.68]	0.195

Time was centered on the last day of the intervention period. Baseline fluid intake was centered on its mean. CI: confidence interval. Model information: *n* participants = 57; observations = 1272; intraclass correlation coefficient (ICC) = 0.28; residual (within-resident) variance (σ^2^) = 100,613.38; between-resident variance of the random intercept (τ_00_ ID) = 38,684.27; marginal R^2^ = 0.320; conditional R^2^ = 0.509.

**Table 4:** Individual assessment of baseline characteristics associated with changes in fluid intake during follow-up.

Characteristics	Estimate (β)	95% CI	*p*-Value
Gender (Man)	68.36	[−44.66, 181.27]	0.228
Nutritional status (Malnutrition)	−101.43	[−251.48, 48.68]	0.178
Pressure ulcers (Risk)	18.18	[−136.53, 172.91]	0.815
Urinary incontinence	76.03	[−111.28, 263.39]	0.419
Dysphagia	−237.04	[−364.92, −109.20]	**<0.001**
Diabetes	133.00	[20.11, 245.73]	**0.019**
Congestive heart failure	110.96	[−59.88, 281.86]	0.196
Diagnosed dementia	−81.12	[−201.18, 39.17]	0.179
Stroke	26.61	[−114.98, 168.31]	0.708
Kidney disease	−57.31	[−180.40, 66.09]	0.354
≥4 chronic diseases	−48.24	[−249.85, 153.30]	0.633
Polypharmacy	−11.19	[−209.43, 186.93]	0.910
Dehydration	144.87	[−78.14, 366.60]	0.195

CI: confidence interval.

**Table 5:** Final mixed-effects linear regression model including baseline characteristics associated with changes in fluid intake during follow-up.

Predictors	Estimate (β)	95% CI	*p*-Value
(Intercept)	19.23	[−86.77, 125.27]	0.719
Group [Intervention 2]	−348.09	[−467.05, −229.17]	**<0.001**
Time	−0.41	[−4.15, 3.34]	0.831
Baseline fluid intake	−0.66	[−0.79, −0.53]	**<0.001**
Functional dependence	−13.19	[−126.90, 100.43]	0.817
Dysphagia	−230.55	[−352.29, −108.97]	**<0.001**
Diabetes	125.08	[23.96, 226.16]	**0.014**
Group [Intervention 2] × Time	−3.26	[−8.20, 1.69]	0.196

Time was centered on the last day of the intervention period. Baseline fluid intake was centered on its mean. CI: confidence interval. Model information: *n* participants = 57; observations = 1272; intraclass correlation coefficient (ICC) = 0.21; residual (within-resident) variance (σ^2^) = 100,602.28; between-resident variance of the random intercept (τ_00_ ID) = 26,976.24; marginal R^2^ = 0.376; conditional R^2^ = 0.508.

## Data Availability

The data presented in this study are available from the corresponding author upon reasonable request due to ethical and confidentiality restrictions, as explicit consent for data sharing was not obtained from the participants. In addition, because the study was conducted in a single care home with a small sample in a limited geographical area, there is a potential risk of indirect identification of both the institution and the participants.

## Abbreviations

The following abbreviations are used in this manuscript:

SAR	Socially Assistive Robot
